# Full deflection profile calculation and Young’s modulus optimisation for engineered high performance materials

**DOI:** 10.1038/srep46190

**Published:** 2017-04-11

**Authors:** A. Farsi, A. D. Pullen, J. P. Latham, J. Bowen, M. Carlsson, E. H. Stitt, M. Marigo

**Affiliations:** 1 Applied Modelling and Computation Group, Department of Earth Science and Engineering, Imperial College London, South Kensington Campus, London SW7 2AZ, United Kingdom; 2 Department of Civil and Environmental Engineering, Imperial College London, South Kensington Campus, London SW7 2AZ, United Kingdom; 3 Department of Engineering and Innovation, The Open University, Walton Hall, Milton Keynes MK7 6AA, United Kingdom; 4 Johnson Matthey, P.O. Box 1, Belasis Avenue, Billingham, Cleveland, TS23 1LB, United Kingdom

## Abstract

New engineered materials have critical applications in different fields in medicine, engineering and technology but their enhanced mechanical performances are significantly affected by the microstructural design and the sintering process used in their manufacture. This work introduces (i) a methodology for the calculation of the full deflection profile from video recordings of bending tests, (ii) an optimisation algorithm for the characterisation of Young’s modulus, (iii) a quantification of the effects of optical distortions and (iv) a comparison with other standard tests. The results presented in this paper show the capabilities of this procedure to evaluate the Young’s modulus of highly stiff materials with greater accuracy than previously possible with bending tests, by employing all the available information from the video recording of the tests. This methodology extends to this class of materials the possibility to evaluate both the elastic modulus and the tensile strength with a single mechanical test, without the need for other experimental tools.

Significant recent advancements achieved in manufacturing technology, including the new opportunities made possible by additive manufacturing (3D printing) have opened the doors to a range of new engineered materials with complex architectures and enhanced mechanical properties^1^,^2^,^3^,^4^. High-performance mechanical components that exhibit high strength and stiffness have found useful applications in different fields in medicine, engineering and technology: stronger and more reliable artificial bones can improve the lives of bone cancer patients; more resistant catalytic pellets can reduce costs in the production of hydrogen, ammonia and other industrial chemicals; the same applies to the pellets that make up the nuclear fuel in the core of nuclear power plants. In all of these applications, computational tools and numerical simulations have become essential as effort is focussed on process optimisation^5^,^6^,^7^,^8^,^9^. However, the mechanical properties of this kind of materials are significantly affected by their microstructural design and by the sintering process^10^,^11^, (i.e. the geometry and scale of the finished product, type of powder, compaction, extrusion or printing tools, firing time and temperature). Consequently, it is critical that characterisation of the mechanical behaviour of these novel materials is carried out rigorously and on specimens representative of the final product.

As these stiff and strong materials are generally considered to be brittle elastic^12^, Young’s modulus and tensile strength are two of the main parameters needed in order to predict the mechanical behaviour of systems made from such components. A variety of methods to determine the Young’s modulus can be found in the literature: they can be broadly categorised as either dynamic or quasi-static. Dynamic methods (such as ultrasonic, prism resonance and impulse excitation tests) typically use knowledge of the density and geometry of specimens, together with measurements of a dynamic response to a transient or cyclic loading. Quasi-static methods (such as micro- and nanoindentations, direct compression and tension tests, flexural tests) use the deformation or strain response of a specimen to a series of constant loads or a continuous loading applied at a low rate such that inertial effects can be ignored.

Dynamic methods such as resonance frequency methods and ultrasonic tests on the one hand need very little sample preparation and the inferred elastic constants can be related to the static Young’s moduli^13^. Because of their sintering processes, many of these materials can only be cast into small pellets, and wave scattering and difficulties in the alignment of tiny samples with the emitting and receiving beams can sometime constitute a disadvantage in employing these methods.

Nanoindentations are also widely employed to characterise the elastic properties and the mechanical behaviour of materials at the micro- and nanoscale^14^,^15^. They require high-resolution testing equipment and in some cases also time and particular tools for the sample preparation (i.e. grinding, polishing, etc.). In the context of ceramics, this technique is particularly well suited when applied for the characterisation of the properties of thin films and small samples such as ceramic coatings and small structural features. To increase the accuracy of the tests, the indentations are generally repeated in a certain area of the sample. However, the resulting Young’s modulus distributions could be affected by the surface condition of the specimen, the sharpness of the indenter and other test conditions. In addition, the inferred Young’s modulus reflects the features of the indented portion of the tested material (such as the external surface) and may not be representative of the structural behaviour of the sample at the macroscale.

When possible, other quasi-statically determined parameters such the elastic modulus from uniaxial compression and the flexural modulus, are generally preferred because they require more conventional and simpler testing machineries and specimen preparation. In addition, they are also generally considered to be more representative of in-service loading conditions^16^. One of the main advantages that these tests offer, compared to dynamic methods, is that they can provide an estimate for both the strength and the stiffness of the material with a single experiment. While the strength is just a function of the sample geometry and the load at failure, Young’s modulus is also related to the relation between the applied loads and the corresponding deformations in the sample (i.e. the recorded displacements). Since the deformations of highly stiff materials are inevitably small and therefore not easy to determine with a high level of confidence, the estimated Young’s modulus can be significantly affected by experimental errors. Bending tests, compared to uniaxial compressions, have the advantage of emphasising the response of the tested sample to the applied loads, effectively increasing the measurable deformations of the sample during the test.

The EN 843-2:2006 Section 4^17^ describes how the three-point bending test can be employed to evaluate Young’s modulus using either the test rig cross-head displacement (Method A.1), displacement transducers (Method A.2) or strain gauges (Method A.3). The Method A.1 is generally not applicable because of the effects of standard rig self-compliance in a three-point bending test for the case of particularly stiff sample bars. This is caused by the great ratio between the applied load and the relatively small deformations of the samples before failure. A typical example for such stiff materials of load-displacement curve of the three-point bending test, shown here for a porous alumina sample, is given in Fig. 1. About 80% of the total displacement recorded by the rig (continuous line) is not representative of the actual alumina beam deflection but is likely to be due to load-frame compliance, based on the expected specimen displacement (dashed-line). Although EN 843-2:2006 Section 4.4 suggests to repeat the test on a thick metal or ceramic bar to estimate the rig self-compliance, the accuracy of the estimated Young’s modulus relies on the assumptions that the thick sample is perfectly rigid and that the rig is responding with the same self-compliances to the transmitted loads, and this might not be true for all the actuators.

Methods A.2 and A.3 are also not convenient when the specimens are too small to allow a standard transducer or strain-gauge to be accurately installed and employed with a high level of confidence. Digital Image Correlation (DIC) is a non-contact optical method that can be employed for monitoring displacements and deformations from sequences of images^18^,^19^,^20^,^21^. This technique can be used to emulate either the arrangement of transducers or the strain gauge as described in Method A.2 and A.3 respectively. Optical observation methods make it possible to eliminate the test rig compliance by tracking discrete optical targets, e.g. discrete markers placed on the two supports and the loading plate in the three-point bending test. It is also possible to average the displacements of a number of random markers in an area expanded around the discrete targets to increase the accuracy of the tracked displacements. Although relatively easy to implement, these methods of harnessing optically recorded data only use data from limited regions of each image, effectively discarding most of the available information. This opens an opportunity for the development of a more reliable method that uses all of the available image deformation data during bending tests, leading to higher levels of accuracy in deflection calculation and therefore in Young’s modulus evaluation.

## Results

### Full deflection profile calculation

**Figure 2:**
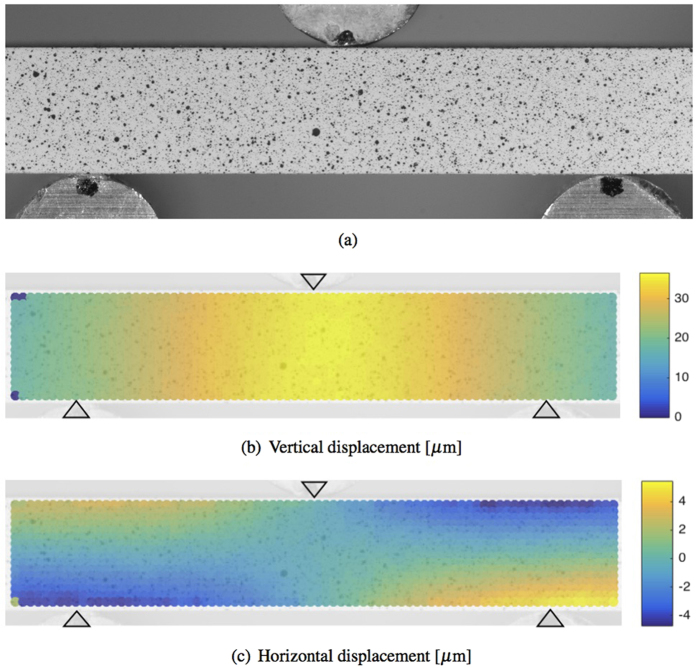
(**a**) An example of the frames used to extrapolate the displacement field of the beam during a three point bending test, (**b**) the vertical displacement field d_v_ and (**c**) the horizontal displacement field d_h_ before failure.

**Figure 3:**
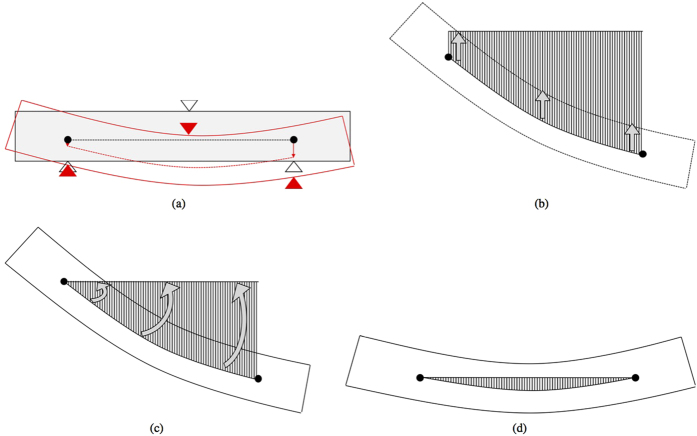
(**a**) Sample configuration before (black) and during the test (red). Schematic representation of the average vertical displacement correction by applying (**b**) a rigid vertical translation and (**c**) rotation to obtain (**d**) the corrected full deflection profile of each frame.

**Figure 4:**
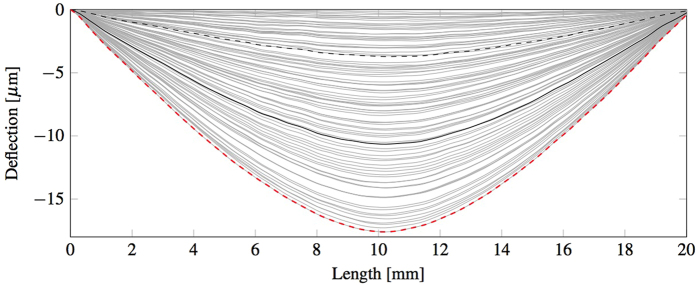
Typical sequence of deflection profiles before failure (grey). In particular, the profile at 20% (dashed black), 60% (black) and 100% (dashed red) of peak load are shown.

The samples were prepared and tested as described in the Methods sections. The displacement field of the beam (Fig. 2) is discretized in a regular grid and for each frame the DIC software calculates the vertical (d_v_) and horizontal (d_h_) displacement of each cell in the grid. The mean vertical displacement of the bar along the horizontal axis w’(x) is calculated for each frame by averaging the displacement of the corresponding cells through the height of the beam, as shown in equation (1). The averaged vertical displacement of the beam is then corrected by fixing the vertical displacement of the left w(x_l_) and right w(x_r_) support to zero. This is done by applying to the averaged vertical displacement the rigid translation *C* and rotation *φ*, as defined in equations (2) and schematised in Fig. 3. The effects of the rotation on the horizontal axis can be neglected since they are much smaller than the cell discretisation. The corrected deflection profile can be calculated for each frame of the recorded experiment with equation (3) and an example is shown in Fig. 4.


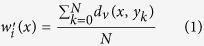



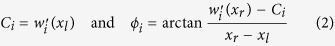



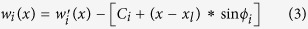


### Young’s modulus calculation

The sequences of beam deflection profiles were synchronised with the load histories recorded by the test rig transducer, resulting in a value of applied load for each profile. Assuming that the beam cross sections remain planar and normal to the deformed axis of the beam, the theoretical vertical displacement profile (w_EB_) associated with the applied load can be express as a function of the Young’s modulus (E) of an equivalent linear-elastic isotropic and homogeneous beam with a defined geometry. In equation (4) the theoretical vertical displacement is defined as a function of the location (x) and E, whereas the moment of inertia (I) and the span between the two supports (s) are two constants that are fixed with the geometry of the tested beam. A single value of Young’s modulus can then be determined for each frame, index =*i*, by minimising the sum of the squares of the differences between the theoretical and the corresponding experimental deflection (least squares) along the entire length of the beam between the supports. By repeating the minimisation shown in equation (5) for each frame, it is possible to determine a series of intermediate Young’s moduli (*E*_*i*_) that best represent the deflection of the beam for each applied load at each frame. These intermediate moduli can then be used to define a single value of Young’s modulus that best represents the linear stress-strain relationship for the tested material over any selected range of applied load. The range between the 20% and 80% of the peak load was selected to define a single value of Young’s modulus for each specimen. The intermediate moduli were therefore converted to their corresponding values of deflection at mid-span and a linear least squares regression was performed on the variable of deflection for the applied load in the defined range. A value of Young’s modulus was then determined for each specimen with equation (6), where *H* is the height of the sample and *m* is the slope of the corresponding line of best fit.


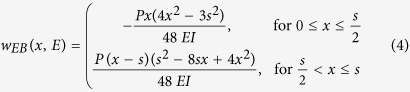



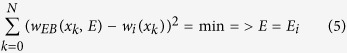



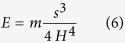


### Uncertainty and optical distortions

**Figure 5:**
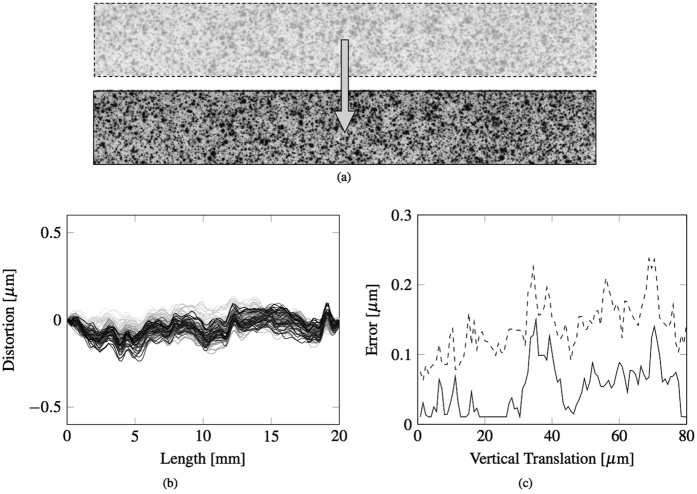
(**a**) Beam configuration before and after applying a rigid body motion. (**b**) Optical distortion in the horizontal profile of the vertical displacement of the speckle panel during the experiment. (**c**) The trend of the estimated maximum errors for each frame.

The proposed approach relies on the assumption that the plane of the target is not displacing significantly in the direction normal to that plane, that is, toward or away from the camera, which would falsely indicate expansion or contraction respectively. For these experiments, this can be said to be true, since the maximum in-plane displacements in the direction of loading, which would be dominant, were of the order of only 1 or 2 pixels. To estimate the error for a possible optical distortion, an independent experiment was considered. A constant vertical displacement was applied to an identical speckle panel connected to the top punch of the three-point bending rig, as shown in Fig. 5(a). The experiment was performed in displacement control, with a cross-head velocity of 0.4 mm/sec that on average corresponds to a vertical displacement of 0.8 *μ*m per frame. Since the maximum vertical displacement before correction experienced on average by the bars before failure was generally 30–40 *μ*m, depending on the tested sample, e.g. in Fig. 2, the error was conservatively evaluated over 100 frames that correspond to a total vertical displacement of 80 *μ*m, which is twice the typical displacement range of the tested sample. The same procedure applied to the beam samples was employed to calculate the horizontal profile of the vertical displacement of the speckle panel. In Fig. 5(b) the corrected beam deflection is shown for each considered frame. Since the panel is subjected to a rigid vertical translation with no deflection, the estimate error for each location of the deflection profile calculated with the proposed methodology can be defined as the maximum absolute value of the deflection profile in each frame. The estimated error, as shown in Fig. 5(c) (dashed line), tends to a value between 0.1 and 0.2 *μ*m. This error is too conservative when the deflection profile is employed for the calculation of Young’s modulus. In this case all the locations of the deflection profile are instead compared to the theoretical deflection in the optimisation process. The estimate of the deflection error in this case can then be defined as the maximum deflection (halfway between the two supports) of the best interpolation curve given by the Euler-Bernoulli theoretical deflection profile. In this case the estimated error, as shown in Fig. 5(c) (continuous line), is lower than the previous. The maximum value of this estimated error tends to 0.1 *μ*m and corresponds to a vertical displacement similar to the maximum before failure in the actual test. Since the Young’s modulus for any applied load is a linear function of the maximum (middle) value of the beam deflection profile, and that this value varies between 15 and 20 *μ*m, depending on the tested sample, the relative error caused by optical distortions on the last estimates of the Young’s modulus calculated before failure is between 0.5% and 0.7%.

### Precision and accuracy

**Figure 6:**
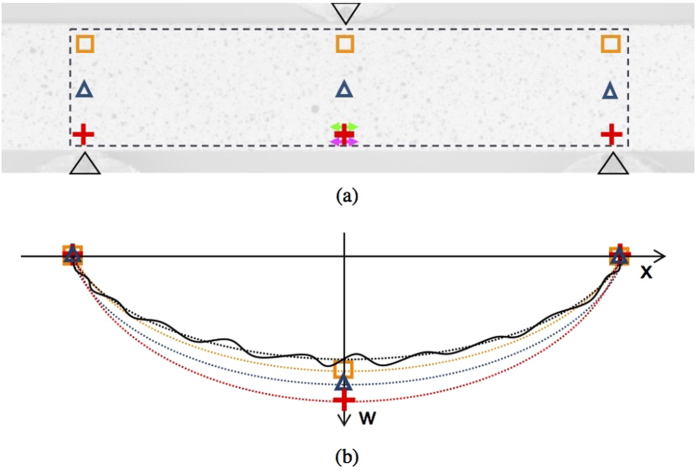
(**a**) Representation of the points from which the vertical displacements are calculated with a standard method using three different sets of virtual displacement gauges (orange squares, blue triangles and red crosses) and the area used with the proposed methodology (black dashed line). The two arrows (green and magenta) represent the locations of two virtual strain gauges. (**b**) Schematic representation of the different determination of the vertical displacements with the standard method from three data points at two different locations (yellow, blue and red) and with the proposed methodology from the best interpolation of the full deflection profile (black).

What follows is a comparison between the proposed methodology and the standard approaches suggested in EN 843-2:2006, employing both displacement and strain data extracted at discrete locations from the full-field DIC deformation data. As shown in Fig. 6(a), we compare the following:

- The proposed methodology;
- Three virtual displacements (mid-span and at both supports, three sets placed at different heights on the viewed surface of the beam;
- Two virtual strain gauges placed close to the lower surface of the beam (corrected to indicate a value of strain on the lower surface).

For the purposes of this comparison, data sets from a single representative bending test have been used. The algorithm suggested in EN 843-2:2006 for displacement transducers is equivalent to calculating the Young’s modulus by Euler-Bernoulli bending theory, but using only the maximum vertical displacement at mid-span relative to the average vertical displacement at the two supports, as shown in Fig. 6(b).

The proposed method employs the entire vertical displacement data set, covering the whole observed surface of the beam, in order to obtain the full deflection profile for the specimen. This deflection profile is corrected to eliminate rigid-body translations and rotation, then analysed to determine the Euler-Bernoulli deflection curve of best fit, as shown in Fig. 6(b). By repeating this process for each frame, it is possible to determine a series of intermediate Young’s moduli that best represent the deflection of the beam for each applied load at each frame. These intermediate moduli can subsequently be used to define a single value of Young’s modulus that best represents the linear stress-strain relationship for the tested material. The employment of a larger set of displacement data for the determination of the beam bending and of a more sophisticated method to account for the rigid-body translations and rotation are the key features that allow the proposed method to have a higher level of precision compared to the standard method with three displacement transducers.

In order to compare the levels of precision of the two methodologies, the intermediate Young’s moduli determined using the proposed methodology were converted back to their corresponding values of deflection at mid-span, for each applied load. In this way it is possible to compare the force-deflection curve from the proposed methodology (i.e. full-field) to those obtained from the DIC-equivalent standard method based on sets of virtual displacement gauges placed at different heights of the beam. The values of force and deflection at 20% and 80% of the peak load were then used to estimate the Young’s modulus from the three curves following the suggested procedure in the EN 843-2:2006 Section 4.

**Figure 7:**
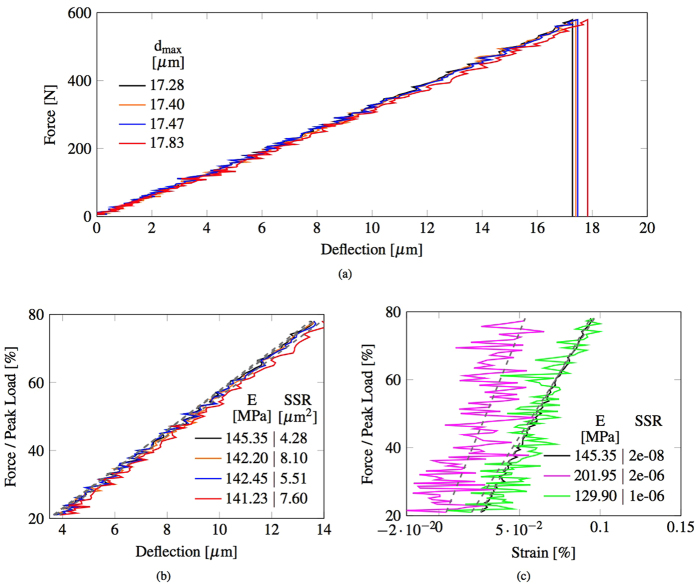
(**a**) Comparison of the force-deflection curves calculated with the standard method from the three sets of three data points (orange, blue and red) and with the proposed methodology from the best interpolation of the full deflection profile (black). (**b**) Subset of the data shown in (**a**) with force as a percentage of the peak load and limited to the range 20% and 80%. The corresponding linear trends (grey dashed lines) obtained from the Young’s modulus extrapolated from the two curves at 20% and 80% of the peak load are also plotted for each set. (**c**) Comparison of the force-strain curves from the two virtual strain gauges and from the proposed methodology. The corresponding linear trends (grey dashed lines) obtained from the Young’s modulus extrapolated from the three curves at 20% and 80% of the peak load are also reported in the graph.

The force-deflection curves shown in Fig. 7(a) suggest that the standard method can be sensitive to the choice of location of the virtual gauges on the surface of the beam, in particular to whether the virtual gauges are located near the inner or outer arc of the deflecting beam. This variability in the results from the standard method indicates a lower precision, as it generates in our examples three values of Young’s modulus that differ by more than 3%, with a lower value obtained in the location of lowermost positions, which are analogous to the transducer positions indicated in the EN 843-2:2006 Section 4. In the supporting reference for the EN 843-2:2006^22^ it is noted that the standard quasi-static flexural method generally produces lower values than other standard methods on ceramic materials. Instead, the proposed methodology employs the data from the whole surface of the beam, from the inner to the outer arc, eliminating the variability due to the choices of the virtual transducer locations. The proposed methodology generates a higher Young’s modulus than those determined by virtual displacement gauges (using DIC data) and adopting standard calculation methods. This suggests that the proposed methodology achieves higher accuracy and may be removing a potential bias towards lower values that is likely to be imposed by the standard quasi-static flexural method.

In Fig. 7(b) the force has been normalised with the peak load and the data range has been limited to 20% to 80% of the peak load (within the 10–90% range indicated by EN 843-2:2006). Also plotted are the trend lines as determined by the standard analysis method, which only considers values at two operator-selected evaluation points (for example, 20% and 80% of the peak load). A quantification of the level of precision can be obtained by calculating the sum of squared residuals (SSR) between values indicated by the linear trend and the actual deflection data, which represents the measurement deviation from the value indicated by that trend. The proposed full-field methodology has a lower SSR (tabulated within Fig. 7(b), 4.28 against 5.51, 7.60 and 8.10 *μm*^2^), and therefore a higher level of precision. The intermediate Young’s moduli from the proposed methodology were also used to back-calculate the corresponding values of horizontal strain on the lower surface of the specimen at mid-span for each value of applied load. Comparable values of horizontal strain were also extracted directly from the full-field DIC deformation data, at two discrete locations just above the lower edge of the observed surface. These were each corrected to an equivalent value at the lower surface. The resulting force-strain data (from 20% to 80% of peak load) is plotted in Fig. 7(c). The values of force and strain at 20% and 80% of peak load were then used to estimate the Young’s modulus, as suggested in EN 843-2:2006 Section 4. Again, a quantification of the level of precision can be obtained also from the calculation of the SSR between values indicated by the linear trend and the actual strain data. The proposed methodology based on full-field DIC has a SSR that is two orders of magnitude smaller than the ones obtained with the standard method (tabulated within Fig. 7(c) 2.07 10^−8^ against 1.44 10^−6^, and 2.08 10^−6^), and therefore a much higher level of precision.

This comparison has been carried out for an experiment that was recorded with state of the art equipment and therefore only high resolution images were analysed using DIC. It is reasonable to assume that the improvements in precision of the proposed methodology would be more significant for lower resolution images because the entire deformation data set (support to support and over the full height) has been used rather than much smaller subsets of that data representing just a few discrete locations.

## Discussion

**Figure 8:**
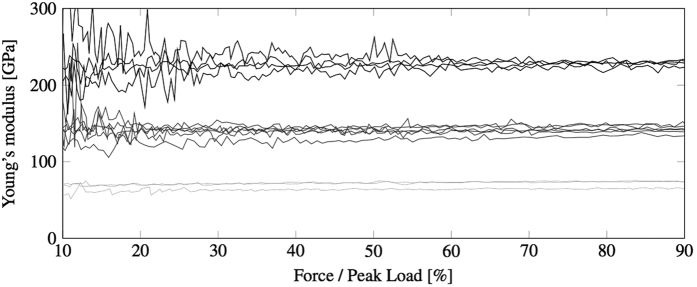
Young’s modulus optimisation from full displacement profiles of the bars of Set 1 (light grey), Set 2 (dark grey) and Set 3 (black).

The proposed methodology was applied to the bending test recordings of 15 samples that were sintered, as described in Methods, to obtain three grades of porosity, with Young’s moduli expected to be in the 60–250 GPa range. In Fig. 8 the estimate of the Young’s modulus of the tested samples is plotted for each frame as each three-point bending test progresses. The optimisation becomes stable after typically forty frames (about 20–30% of the peak load) due to the fact that in the first phase of the experiment, i.e. when the punch makes contact with the sample, the load and deformations are of very low magnitude and more significantly affected by noise in the signal from the transducer and errors in the DIC analysis. The distribution of results is consistent for the three sets of bars and the mean values and standard errors are reported in Table 1.

**Figure 9:**
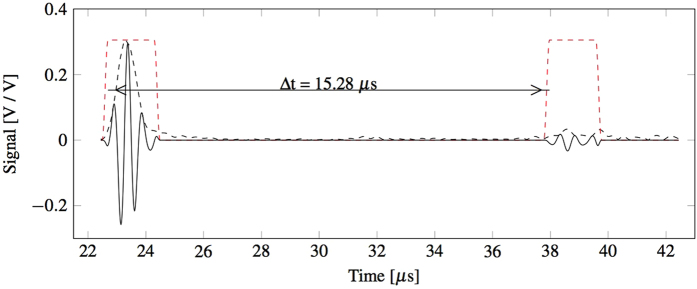
Experimental data of the sound rebound time obtained from an ultrasonic test on a sample from Set 1. In particular, the Hilbert envelope (dashed black), the filter windows (dashed red) and the windowed signal (continuous black).

Bars from the three sets of samples were also tested with ultrasonic techniques. Figure 9 shows the two distinctive sets of peaks recorded for one of the tests recorded for Set1. Since the path between the transducer and the top surface of the sample is travelled twice both by the top and bottom reflected waves, the distance between the two peaks corresponds only to the time that the waves take to travel twice the length of the specimen. Therefore, the velocity of the longitudinal waves propagating through the sample can be expressed as 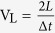. The relation between density (*ρ*), Lamé constants (*λ* and *μ*) of the specimen and the longitudinal wave velocity is 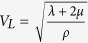
23.

**Figure 10:**
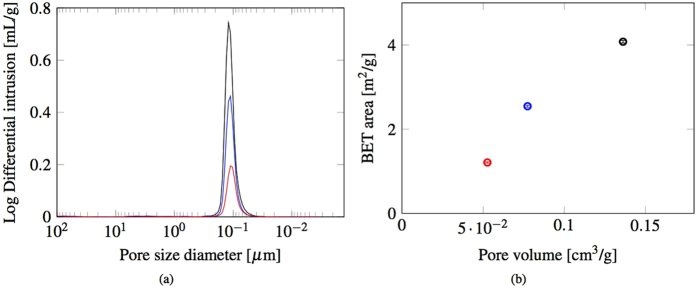
(**a**) Pore size distributions from Hg intrusions on a sample from Set 1 (black), Set 2 (blue) and Set 3 (red) and (**b**) the correlation between the BET area and the pore volume from Hg intrusions.

**Figure 11:**
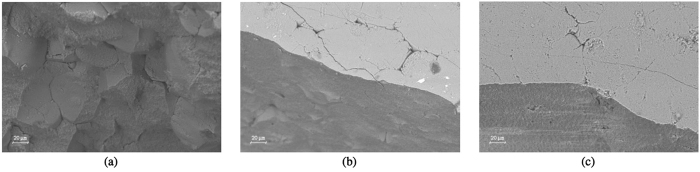
FESEM images of the fracture surfaces of bar fragments showing the microstructural failure mode for the three sets of samples. The scanning of the fracture surface of a sample from Set 1 shows a fracture surface with an inter-granular morphology (**a**), whereas the images from Set 2 (**b**) and Set 3 (**c**) show fracture surfaces (below) and the external surface of the sample (above), without visible grain boundaries.

This relation is only valid for a wave propagating through a homogeneous elastic medium. The microstructure of the three sets of samples was therefore investigated with Mg intrusion, BET adsorption and FESEM, as described in Methods. Figure 10(a) shows that the dominant pore diameter for Sets 1–3 is in approximately 100 nm. There is a slight decrease in the mean pore diameter as the firing temperature increases. Figure 10(b) shows that the porous volume available for Hg intrusion decreases with increasing firing temperature. Figure 10(b) also displays the anticipated correlation between the internal surface area of the sample, measured using BET adsorption, and the pore volume. The FESEM results show that the difference in the microstructure within the samples is not significant. The FESEM analyses of the fragments from the the bending tests, show some differences in the microstructural behaviour of the three sets of bars. The fractured sample from Set 1 exhibits mostly inter granular fractures, as shown in Fig. 11(a). The fragments from Set 2 and 3 on the other hand reveal predominantly intra granular fractures, as shown in Fig. 11(b,c) respectively, indicating stronger grain boundaries.

A 25 MHz ultrasonic transducer was employed for the tests and the measured lower wave velocity measured was above 5,000 m/s. Therefore the wavelength of the transmitted waves is above 200 *μ*m. The high ratio between the wavelength and the average pore size of the specimens (0.1 *μ*m) allows to trait the samples as homogeneous media with respect to the transmitted ultrasonic waves. By substituting in the previous equation of the wave velocity the relations between the Lamé constants and Young’s modulus 
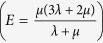
, Poisson’s ratio 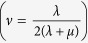, it is possible to express the Young’s modulus of the tested specimen as a function of the longitudinal wave velocity, Poisson’s ratio and bulk density: 
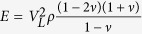
. Since the test apparatus did not allow to transmit transverse wave through the specimen, the Poisson’s ratio could not be experimentally estimated. Previous publications on ultrasonic tests on porous alumina samples report a value of 0.17 for the Poisson’s ratio, assuming that it is ‘approximately independent’ of porosity^24^,^25^. In more recent publications^26^,^27^ the relation between Poisson’s ratio and porosity of alumina samples has been experimentally investigated, showing that samples with similar porosity to the ones employed in the present work have Poisson’s ratio in the 0.17–0.20 range. The Poisson’s ratio was therefore assumed to be 0.17 as its variability affects the estimated Young’s moduli by less than 3%.

**Figure 12:**
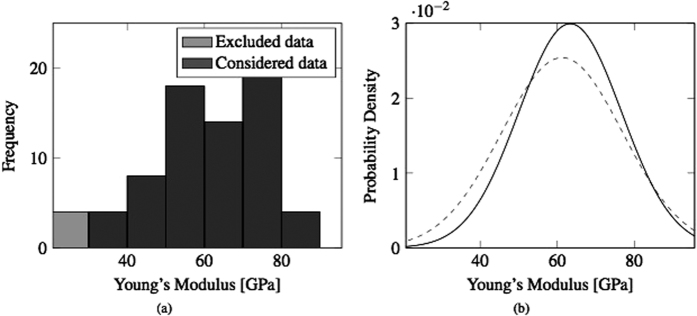
(**a**) Histograms, (**b**) original (dashed) and corrected (continuous) normal probability distributions of the Young’s modulus estimations by nanoindentations for a sample of Set 1.

One specimen from each set of samples was also tested with nanoindentations, as described in Methods. For each bar, one hundred indentations have been performed for statistical correction to minimize the experimental error. The histograms and normal probability distributions of the Young’s modulus were estimated with one hundred nanoindentations on the surface of a sample. A correction was performed by excluding the experimental results that were 50% either lower or higher than the average value of the entire distribution. The results from the indentations on a sample from Set 1 are shown in Fig. 12.

**Figure 13:**
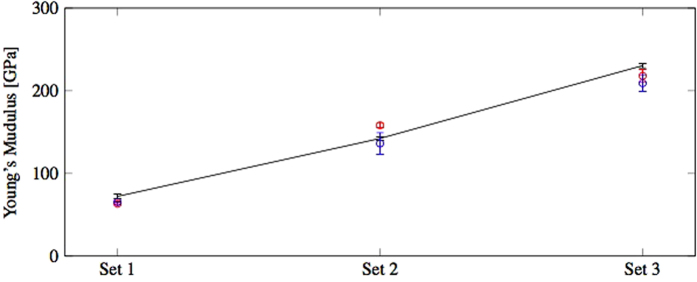
Comparison of the Young’s moduli from the proposed methodology (black), ultrasonic tests (blue) and nanoindentations (red).

The distributions of the extrapolated Young’s moduli from the ultrasonic tests and nanoindentations are in agreement with the ones from the proposed methodology and the mean values and standard errors are also reported in Table 1. A comparison of the results from the three tests is also shown in Fig. 13.

### Concluding remarks

A simple and accurate methodology for the calculation of the full deflection profile for bending tests and an optimisation algorithm for the characterisation of the Young’s modulus have been presented. The experimental results that have been shown in this paper corroborate the capabilities of this methodology to evaluate the Young’s modulus of highly stiff materials with greater accuracy than previously possible with bending tests, by correcting the errors induced by self-compliances of test rigs employing information from the video recording of the test. Furthermore, it extends to this class of engineered high performance materials the possibility to evaluate both the elastic modulus and the tensile strength of the tested sample (the main parameters that are needed to simulate systems incorporating brittle elastic materials) using a single mechanical test. This alone constitutes a great advancement with respect to the standard tests that are normally employed for this class of materials. Although the experiments reported refer to prismatic alumina bars, the methodology can be extended to cylindrical or other extruded shapes or even samples with higher aspect ratio that exhibit brittle elastic behaviours. Further research will need to be undertaken to extend the proposed study to materials with more complex constitutive models, such as specimens that exhibit significant plastic deformations before failure. A similar methodology to the one presented in this paper can employ mechanical simulation software tools to perform back analysis of the plastic parameters needed to simulate the performance of structures with elastic-plastic components.

## Methods

### Sample preparation

Three sets of prismatic samples were sintered with a reference alpha-alumina powder with an average granulate size in the 170–210 *μ*m range that was compacted at an initial bulk porosity 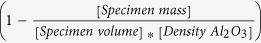 of 0.45 and then fired at 1200 °C, 1300 °C and 1400 °C to obtain three sets of bars with final bulk porosity of 0.36, 0.26 and 0.15 respectively. The geometry and density of the tested samples are reported in Table 2.

### Hg intrusion and BET adsorption

Samples from the three sets of bars were analysed using a MicroActive AutoPore V 9600 mercury intrusion porosimeter and a ASAP 2420 BET adsorption apparatus.

### FESEM

Bars and fragments from the three sets of bars were analysed using a Zeiss ultra 55 field emission electron microscope equipped with in-lens secondary electron and backscattered detectors. The samples were carbon coated prior to analysis to provide a conductive layer for charge dissipation. The high-resolution low-accelerating voltage imaging was performed with an accelerating voltage of 1.6 kV, aperture of 20–30 *μ*m and a working distance of 2–3 mm. The low-resolution general imaging was performed with an accelerating voltage of 20 kV, aperture of 30–60 *μ*m and a working distance of 7–8 mm

### Three-point bending test

#### Experimental setup

The three-point bending test-fixture consists of two supports and a loading platen mounted on an instrumented test rig. The parallel pair of semi-cylindrical supports of 6 mm in diameter were set 20 mm apart and mounted on a monolithic cylinder that was placed centrally on the stationary base of the test rig (Instron model 5984 electromechanical test frame). An opposing semi-cylindrical loading platen of 7.2 mm in diameter was mounted centrally on the vertically-moving crosshead of the test rig, below the load-cell. The experiments were performed in displacement control, with a crosshead velocity of 0.5 mm/min. The test rig control software (Instron Bluehill 3) recorded load and displacement during each experiment, at 0.1 second intervals. The experiments were recorded with a high-speed video-camera (Vision Research Phantom v12.1 monochrome, maximum capture rate 6000 frames/second at full-resolution of 1280 by 800 pixels, fitted with a 100 mm macro lens). The optical axis was set normal to the speckled side-face of the specimen. A high-speed video camera was used in order to potentially capture the fracture event at the end of the test, and to allow for the possibility of higher rates of loading in future experiments without a change of equipment. However, the high-speed capability is not required for the proposed methodology but the monochrome 12-bit (4096 intensity levels) sensor of this camera is well suited to digital image correlation, compared with (for example) a 24-bit (3 × 256 levels) colour sensor.

#### Digital image correlation analysis

Prior to testing, one side-face of each specimen had a random speckle pattern applied. This was achieved by spraying the white surface of the specimens with a black ink resulting in an even but random distribution of marks (“speckles”). This pattern is captured in a series of images and used by the DIC software to track the surface deformations. Digital image correlation analyses were performed on the image sequences recorded by the high-speed camera, using commercially available software (LaVision DaVis/Strainmaster)^28^. For each experiment, each image in the sequence was compared with the same initial or reference image representing the undeformed specimen. The Region Of Interest (ROI) was the full width of the image (1280 pixels) by typically 220 pixels high, depending on the height of the specimen. Since each image was a single view of the side-face of the specimen, only in-plane deformations of the ROI were produced as an output of the DIC analysis. The primary output of a 2D DIC analysis is a matrix with the vertical (d_v_) and horizontal (d_h_) displacement values at each location in a regular grid, based on the initial pixel locations or indices of the reference image. For these experiments, the beam specimens were aligned parallel to the image edges, so each x value represented a horizontal position within the specimen and each y value represented a vertical position within the height of the specimen. The d_v_ and d_h_ data for each image was exported from the LaVision software and further analysed using Matlab^29^ for the deflection profile calculation and the Young’s modulus optimisation.

### Ultrasonic test

The ultrasonic equipment consisted of a 25 MHz piezoelectric transducer, which emits and receives longitudinal waves through a bar sample immersed in a water bath, an amplifier and computer that records and processes the signals. Each tested specimen, after being coated with an impermeable thin layer, was placed on a thin metal support that separates the specimen bar from the bottom wall of the water tank and maintains the sample vertical and parallel with the transducer beam. A partially motored apparatus is then employed to align the ultrasonic beam with the longer dimension of the sample bar. This was done to maximise the length that the waves have to travel before and after being reflected by the bottom surface of the sample so that the recorded signals have two distinctive peaks that represent the top and bottom reflections.

### Nanoindentations

The nanoindentation apparatus has maximum load of 400 mN, load noise of <1 *μ*N, maximum depth of 1,000 nm, and depth noise of <0.2 nm. A Berkovich diamond indenter with tip radius of <3 nm has been used to indent the specimen. Each indentation test is performed within 240 s, including a 30 s holding time at the peak load. The testing temperature is maintained within the range of 20–22 °C to reduce thermal drift. The elastic moduli were measured using the Oliver-Pharr method^30^.

## Additional Information

**How to cite this article**: Farsi, A. *et al*. Full deflection profile calculation and Young’s modulus optimisation for engineered high performance materials. *Sci. Rep.* **7**, 46190; doi: 10.1038/srep46190 (2017).

**Publisher's note:** Springer Nature remains neutral with regard to jurisdictional claims in published maps and institutional affiliations.

## Figures and Tables

**Figure 1:**
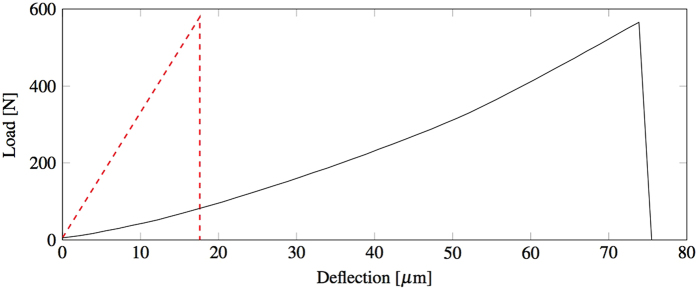
Effects of the rig self-compliance in the three-point bending tests: expected (dashed red) and recorded (black) load-displacement curve.

**Table 1:** Mean values and standard errors of the Young’s moduli from this optimisation methodology, ultrasonic tests and nanoindentations.

Set	Optimisation method	Ultrasonic test	Nanoindentations
	[GPa]	[GPa]	[GPa]
1	72.22 ± 2.75	65.45 ± 0.83	63.36 ± 1.59
2	142.29 ± 2.13	136.22 ± 12.98	158.34 ± 3.11
3	230.43 ± 2.56	209.11 ± 10.24	218.02 ± 7.92

**Table 2:** Average of the measured dimensions and bulk density of the tested prismatic specimens.

Set	L	H	Bulk density
	[mm]	[mm]	[g/cm^3^]
1	40.20 ± 0.01	4.84 ± 0.01	2.58 ± 2%
2	38.09 ± 0.01	4.64 ± 0.01	3.00 ± 2%
3	36.33 ± 0.01	4.41 ± 0.01	3.25 ± 2%
